# Integrating CT radiomics and clinical data with machine learning to predict fibrosis progression in coalworker pneumoconiosis

**DOI:** 10.3389/fmed.2025.1599739

**Published:** 2025-07-22

**Authors:** Xiaobing Li, Qian Li, Xinyi Xie, Wei Wang, Xuemei Li, Tingqiang Zhang, Li Zhang, Yongsheng Liu, Li Wang, Wutao Xie

**Affiliations:** ^1^Science and Technology Industry Development Center, Chongqing Medical and Pharmaceutical College, Chongqing, China; ^2^Laboratory of Toxicology, The First Affiliated Hospital of Chongqing Medical and Pharmaceutical College, Chongqing, China; ^3^Chongqing Key Laboratory of Prevention and Treatment for Occupational Diseases and Poisoning, The First Affiliated Hospital of Chongqing Medical and Pharmaceutical College, Chongqing, China; ^4^Department of Occupational Disease and Poisoning Medicine, The First Affiliated Hospital of Chongqing Medical and Pharmaceutical College, Chongqing, China; ^5^College of Public Health and Health Management, Chongqing Medical University, Chongqing, China; ^6^Clinical Medicine Department, Medical College, Hebei University of Engineering, Handan, Hebei, China; ^7^Department of Radiology, The First Affiliated Hospital of Chongqing Medical and Pharmaceutical College, Chongqing, China; ^8^Department of Neurology, NHC Key Laboratory of Diagnosis and Treatment on Brain Functional Diseases, The First Affiliated Hospital of Chongqing Medical University, Chongqing, China

**Keywords:** coalworker pneumoconiosis, pulmonary interstitial fibrosis, CT radiomics, clinical features, machine learning, predictive model, multimodal joint model, ROC curve

## Abstract

**Objective:**

This study aims to develop a machine learning (ML) model that integrates computed tomography (CT) radiomics with clinical features to predict the progression of pulmonary interstitial fibrosis in patients with coalworker pneumoconiosis (CWP).

**Methods:**

Clinical and imaging data from 297 patients diagnosed with CWP at The First Affiliated Hospital of Chongqing Medical and Pharmaceutical College between December 2021 and December 2023 were analyzed. Of these patients, 170 developed pulmonary interstitial fibrosis over a 3-year follow-up and were classified as the progression group, while 127 patients showed stable conditions and were classified as the stable group. The patients were divided into a training cohort (*n* = 207) and a test cohort (*n* = 90). Radiomic features were extracted from CT images of lung fibrosis lesions in the training cohort. These features were reduced in dimensionality to construct morphological biomarkers. ML methods were then used to develop three models: a clinical model, a radiomics model, and a multimodal joint model. The performance of these models was evaluated in the test cohort using receiver operating characteristic (ROC) curves and decision curve analysis (DCA).

**Results:**

In the training cohort, the area under the curve (AUC) for the clinical, radiomics, and joint models were 0.835, 0.879, and 0.945, respectively. In the test cohort, the AUC values for these models were 0.732, 0.750, and 0.845, respectively. The joint model demonstrated the highest predictive performance and clinical benefit in both the training and test cohorts.

**Conclusion:**

The multimodal model, combining CT radiomics and clinical features, offers an effective and accurate tool for predicting the progression of pulmonary fibrosis in CWP.

## 1 Introduction

Pneumoconiosis is a chronic, progressive fibrotic lung disease caused by the prolonged inhalation and deposition of occupational dust particles, resulting in diffuse pulmonary fibrosis. It includes a spectrum of conditions linked to exposure to airborne particulates such as asbestos fibers, coal mine dust, and respirable crystalline silica (1, 2). Although pneumoconiosis is recognized as a global occupational health issue, its incidence remains disproportionately high in industrialized nations, where environmental dust exposure is endemic (3). China continues to report the highest number of pneumoconiosis cases annually, with a steadily increasing disease burden despite the implementation of occupational health regulations (4).

Among the various subtypes, coalworker pneumoconiosis (CWP) is one of the most common, attributed to prolonged coal dust exposure in mining environments (5). While CWP shares certain clinicopathological features with other dust-induced lung diseases-such as asbestosis and silicosis-its fibrogenic mechanisms differ due to the unique properties of coal dust (6). For instance, while asbestosis and silicosis involve interstitial lung damage triggered by asbestos fibers and crystalline silica particles, respectively, CWP is marked by the accumulation of coal dust, often compounded by silica contamination, leading to a distinct fibrotic response (7, 8). Despite their etiological differences, all types of pneumoconiosis converge on a common pathological trajectory: progressive pulmonary fibrosis (9).

Pulmonary fibrosis represents the principal driver of morbidity and mortality in advanced pneumoconiosis. It is characterized by the relentless accumulation of extracellular matrix proteins in lung parenchyma, which distorts the normal alveolar architecture and results in irreversible impairment of pulmonary function (10, 11). As fibrosis advances, patients often experience a marked decline in lung function, leading to complications such as pulmonary hypertension, cor pulmonale, and ultimately, respiratory failure (12, 13). Given these severe outcomes, early identification and continuous monitoring of fibrotic progression are essential to improving clinical prognosis and guiding therapeutic intervention (14).

However, early-stage fibrosis in pneumoconiosis typically lacks overt clinical symptoms or radiological markers, making it difficult to diagnose using conventional methods (15). Pulmonary function tests and chest X-rays, though routinely employed in occupational health surveillance, have limited sensitivity for detecting subtle interstitial changes (16). High-resolution computed tomography (CT), on the other hand, provides greater diagnostic accuracy, but its interpretation relies heavily on radiologist expertise, introducing subjectivity and variability in clinical assessments (17, 18).

In recent years, radiomics-a technique involving the extraction of high-dimensional features from medical imaging-has emerged as a promising tool to enhance diagnostic precision and capture latent imaging biomarkers not discernible by the human eye (19). In the context of lung disease, CT-based radiomics has been shown to reflect underlying pathophysiological alterations, including fibrotic remodeling, thereby enabling risk stratification and disease prediction (20). When combined with machine learning (ML), radiomic analysis can be further optimized to create predictive models that identify disease progression with improved accuracy and objectivity (21, 22).

The present study focuses on patients with CWP and aims to develop an interpretable, multimodal ML model to predict pulmonary fibrosis progression. By integrating CT radiomics with clinical parameters, we seek to enhance predictive performance beyond what is achievable with either modality alone. Three distinct models were constructed: a clinical model based solely on laboratory and demographic features; a radiomic model derived from CT feature sets; and a multimodal joint model that fuses both clinical and imaging data. These models were systematically trained, internally validated, and evaluated to determine their relative performance in predicting fibrosis progression.

The clinical significance of this study lies in its potential to offer a non-invasive, reproducible, and objective tool for the early identification of pulmonary fibrosis in CWP. Such an approach could facilitate timely clinical interventions, reduce disease burden, and ultimately improve patient outcomes. Furthermore, this methodological framework may be extended to other occupational lung diseases and interstitial lung conditions, underscoring its broader applicability in respiratory medicine.

## 2 Materials and methods

### 2.1 Demographic data

The present study retrospectively analyzed data from the Pneumoconiosis Diagnosis Center at The First Affiliated Hospital of Chongqing Medical and Pharmaceutical College. A total of 297 male patients with confirmed CWP were enrolled. The diagnosis of CWP was established by a certified expert panel at the Chongqing Prevention and Treatment Center for Occupational Diseases, in accordance with national diagnostic criteria for occupational pneumoconiosis.

All included patients were initially diagnosed with CWP without radiological evidence of pulmonary interstitial fibrosis and were followed for a period of 3 years. During the follow-up, 170 patients developed imaging features consistent with pulmonary fibrosis and were categorized into the progression group, while the remaining 127 patients exhibited no significant radiological progression and were assigned to the stable group.

**FIGURE 1:**
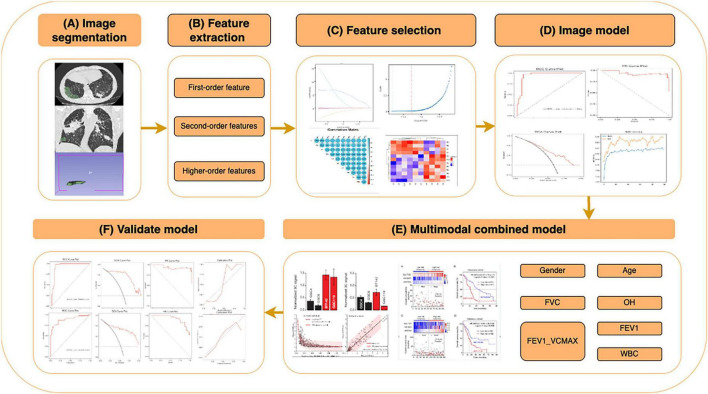
Workflow of the CT radiomics and clinical feature-based machine learning (ML) model for predicting pulmonary fibrosis progression in coalworker pneumoconiosis (CWP). Overview of the workflow for constructing and validating a ML model based on CT radiomics and clinical features, including: **(A)** image segmentation: Regions of interest (ROIs) were segmented from high-resolution CT images, including lung parenchyma and fibrotic regions. **(B)** Feature extraction: radiomics features were extracted and categorized into first-order (intensity-based), second-order (texture-based), and higher-order features. **(C)** Feature selection: relevant features were selected using statistical analysis, correlation heatmaps, and importance ranking to optimize the model’s performance. **(D)** Image model construction: ML algorithms were applied to selected features, and performance was evaluated using metrics such as ROC and PR curves. **(E)** Multimodal combined model: clinical features (e.g., gender, age, FVC, OH, FEV1, and WBC) were integrated with radiomics features to construct a multimodal prediction model, achieving enhanced predictive performance. **(F)** Validation model: the model was validated with independent datasets, assessing metrics such as sensitivity, specificity, and calibration curves.

To facilitate model development and validation, the entire cohort was randomly divided into a training cohort (*n* = 207) and a test cohort (*n* = 90). The training cohort was used for feature selection and model construction, while the test cohort served as an independent validation set. The overall study design, including inclusion criteria and cohort allocation, is summarized in Figure 1.

Inclusion criteria comprised: (1) male patients with a diagnosis of CWP based on the GBZ 70-2015 guidelines, a national Chinese standard titled “Diagnosis of Occupational Pneumoconiosis”, which defines diagnostic criteria for pneumoconiosis using chest radiographs; (2) availability of baseline high-resolution computed tomography (HRCT) imaging and complete clinical data; and (3) no evidence of pulmonary fibrosis at initial presentation. Diagnosis of pulmonary fibrosis during follow-up was based on HRCT criteria outlined in the 2022 American Thoracic Society guidelines for idiopathic and progressive pulmonary fibrosis in adults. Exclusion criteria included incomplete biomarker or imaging data, low-quality CT scans, or co-existing interstitial lung diseases (ILDs) such as tuberculosis-related fibrosis or connective tissue disease-associated pneumoconiosis.

Clinical variables collected for analysis included patient age, cumulative dust exposure time (in hours), pulmonary function test results [forced vital capacity (FVC), forced expiratory volume in the first second (FEV1), FEV1/FVC ratio], and key laboratory indices.

### 2.2 Feature extraction and data pre-processing

Lesion identification: a HRCT images were independently reviewed by a senior thoracic radiologist (over 20 years of experience in occupational lung disease). Radiological signs indicative of fibrotic progression-such as interlobular septal thickening (± subpleural lines), ground-glass opacities (GGOs), reticular patterns (± parenchymal bands), honeycombing with or without traction bronchiectasis, and pleural plaques-were documented. ROI segmentation: lung window images were standardized in grayscale intensity. Target lesions were manually segmented to create regions of interest (ROIs) by a second radiologist with more than 10 years of experience in chest imaging. The segmented volumes were reconstructed into three-dimensional ROIs using 3D Slicer software (version 5.2.2)^1^. A third senior radiologist independently validated the segmentations to ensure reproducibility and anatomical accuracy.

Radiomic feature extraction: a total of 851 radiomic features were extracted from each ROI using the Pyradiomics extension in 3D Slicer. Extracted features included first-order statistics, shape descriptors (2D and 3D), and texture-based metrics, including gray-level co-occurrence matrix (GLCM), gray-level run length matrix (GLRLM), gray-level size zone matrix (GLSZM), and neighboring gray-tone difference matrix (NGTDM).

Data pre-processing: to address sample imbalance in the training set, oversampling techniques (random replication) were applied. All radiomic features were normalized to a range of [0, 1] using the MinMaxScaler function in R (v3.4.1).

### 2.3 Feature dimensionality reduction and construction of radiomic biomarkers

To reduce overfitting risk and enhance model interpretability, dimensionality reduction was performed using the least absolute shrinkage and selection operator (LASSO) regression. This technique imposes an L1 penalty to shrink coefficients of irrelevant or collinear features, thereby improving model generalizability. The initial pool of 851 radiomic features underwent LASSO-based feature selection, yielding a subset of features with the highest predictive value. These features were subsequently used to construct radiomic signatures, referred to as “psychoradiomic signatures” (PS), which encapsulate multi-parametric image information reflective of microstructural changes in lung parenchyma associated with fibrotic progression.

To build the PS, two ML classifiers-logistic regression (LR) and support vector machine (SVM)-were employed. The classification performance was evaluated using the area under the receiver operating characteristic (ROC) curve (AUC), sensitivity, specificity, and calibration plots. A calibration curve was generated to assess agreement between predicted probabilities and observed outcomes, thus evaluating model reliability and potential overfitting.

### 2.4 Construction and validation of the joint model

An integrative model incorporating both radiomic and clinical variables was subsequently developed. The rationale was to exploit the complementary diagnostic information provided by HRCT-derived radiomics and conventional clinical indicators, e.g., pulmonary function metrics (FVC, FEV1, FEV1/FVC). The combined model was trained using an SVM algorithm, given its proficiency in high-dimensional and non-linear classification problems. Prior to training, five-fold cross-validation was employed within the training set to fine-tune hyperparameters, including the kernel function and the regularization coefficient (denoted as C). Model complexity was balanced against predictive performance to prevent overfitting.

Following optimization, the final model was tested on the independent validation cohort (*n* = 90). Model performance was assessed using AUC, sensitivity, specificity, and overall classification accuracy. Calibration curves were again used to verify consistency between predicted risk and actual outcome. Furthermore, decision curve analysis (DCA) was conducted to evaluate clinical utility by quantifying the net benefit of model-assisted decisions compared with treat-all or treat-none strategies.

### 2.5 Statistical analysis

Statistical analyses were conducted using SPSS version 26.0 (IBM Corp., Armonk, NY, USA), R software version 3.4.1, and DecisionLinnc (version 1.0, Nov 2023)^2^. Continuous variables conforming to normal distribution were analyzed using independent-sample *t*-tests, while non-normally distributed data were assessed via the Mann-Whitney U test. Categorical variables were compared using chi-square or Fisher’s exact tests as appropriate. Model diagnostic performance was quantified through ROC-derived metrics including AUC, sensitivity, and specificity. All statistical tests were two-tailed, with a *P* < 0.05 considered statistically significant.

## 3 Results

### 3.1 Comparison of clinical characteristics and CT imaging findings

In the training cohort, a comparative analysis of clinical parameters between the fibrosis progression group and the stable group revealed statistically significant differences in dust exposure duration (DCH), FVC), FEV1, and FEV1/FVC ratio (all *P* < 0.05). These findings suggest that both occupational exposure and lung function metrics serve as important clinical indicators of fibrosis progression in patients with CWP. In contrast, no significant intergroup differences were observed for age or WBC count (*P* > 0.05), indicating limited prognostic value of these variables in this context.

Consistent results were observed in the test cohort: FVC, FEV1, and FEV1/FVC ratio remained significantly different between the progression and stable groups (*P* < 0.05), whereas age and DCH did not reach statistical significance (*P* > 0.05). A comprehensive comparison of clinical variables for both cohorts is presented in Table 1.

**TABLE 1:** Comparative analysis of clinical characteristics between the training and test cohorts in coalworker pneumoconiosis (CWP) patients.

Characteristics	All cohort (297)	Training cohort (*n* = 207)			Test cohort (*n* = 90)			Training vs. Test *P*-value
		Stable (*n* = 86)	Progress (*n* = 121)	*P*-value	Stable (*n* = 42)	Progress (*n* = 48)	*P*-value	
AGE	53.97 ± 7.80	53.94 ± 6.00	54.15 ± 5.99	0.800	53.12 ± 5.23	55.49 ± 6.34	0.0554	0.412
DCH	13.86 ± 7.41	11.15 ± 5.55	15.24 ± 8.61	0.0001*	13.73 ± 5.65	15.31 ± 7.26	0.2508	0.0001*
FVC	90.02 ± 13.10	92.91 ± 7.03	88.50 ± 15.56	0.0066	92.84 ± 7.06	86.35 ± 16.84	0.0168*	0.003*
FEV1	81.52 ± 17.59	89.54 ± 6.21	76.18 ± 20.90	0.0001*	88.10 ± 6.75	75.14 ± 21.09	0.0001*	0.0001*
FEV1/VCMAX	95.69 ± 17.10	107.13 ± 8.66	87.61 ± 17.77	0.0001*	105.85 ± 10.22	87.55 ± 15.79	0.0001*	0.0001*
WBC	7.38 ± 1.69	7.54 ± 1.59	7.28 ± 1.80	0.2673	7.40 ± 1.56	7.52 ± 2.16	0.7751	0.458

Note: *Represent statistically significant.

**FIGURE 2:**
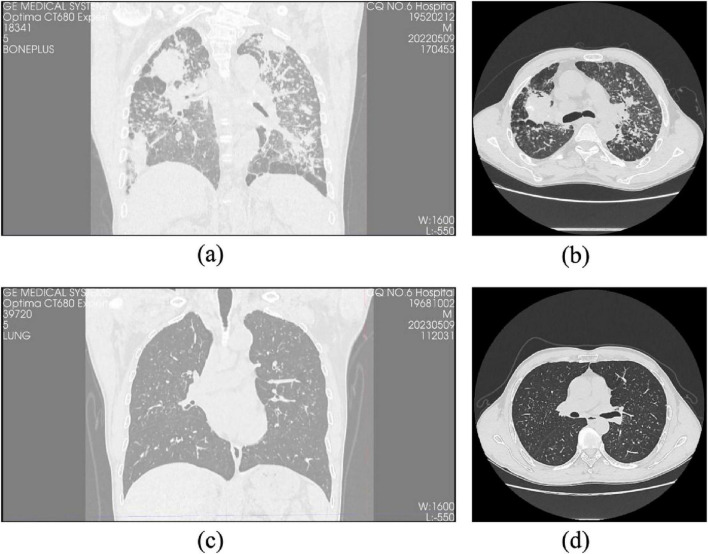
Comparison of HRCT images between progressive and non-progressive CWP patients. **(a)** Coronal HRCT image of a patient with progressive pulmonary fibrosis showing extensive fibrotic lesions and lung structural distortion. **(b)** Axial HRCT images of the same progressive case reveal pronounced interstitial fibrosis with diffuse irregular opacities and honeycombing. **(c)** Coronal HRCT image of a patient with non-progressive pulmonary fibrosis demonstrating relatively preserved lung architecture and limited fibrotic changes. **(d)** Axial HRCT images of the same non-progressive case illustrate minor fibrotic features with a predominance of nodular or reticular patterns and no evidence of honeycombing.

HRCT findings demonstrated excellent inter-observer agreement between two experienced thoracic radiologists in identifying key radiological features associated with pulmonary fibrosis. Principal imaging signs-including interlobular septal thickening, ground-glass opacity (GGO), reticular patterns (“grid shadows”), honeycombing, and pleural plaques-were consistently observed across both patient groups (Figure 2).

Cohen’s kappa coefficients for inter-observer agreement were as follows: 0.786 for interlobular septal thickening, 0.769 for GGO, 0.828 for reticular pattern, 0.814 for honeycombing, and 0.792 for pleural plaques, all demonstrating substantial to excellent agreement (*P* < 0.05). These results, detailed in Table 2, underscore the robustness and reproducibility of the radiological assessment process.

**TABLE 2:** Comparative analysis of CT imaging features between stable and progression groups in coalworker pneumoconiosis.

Feature	Stable (127)	Progress (170)	Statistical test value *X*^2^	*P*-value
**Interlobular septal thickening**				
Yes	72 (56.69%)	95 (55.88%)	0.0849	0.7711
No	55 (43.31%)	75 (44.12%)		
**Ground glass opacity**				
Yes	69 (54.33%)	76 (44.70%)	1.9775	0.1608
No	58 (45.67%)	94 (55.30%)		
**Grid shadow**				
Yes	74 (58.26%)	103 (60.59%)	0.3909	0.4876
No	52 (41.74%)	67 (39.41%)		
**Honeycomb shadow**				
Yes	46 (36.22%)	48 (28.23%)	1.9405	0.1616
No	81 (63.78%)	122 (71.76%)		
**Pleural plaques**				
Yes	31 (24.40%)	56 (32.94%)	1.0986	0.2949
No	96 (75.60%)	114 (67.05%)		

**FIGURE 3:**
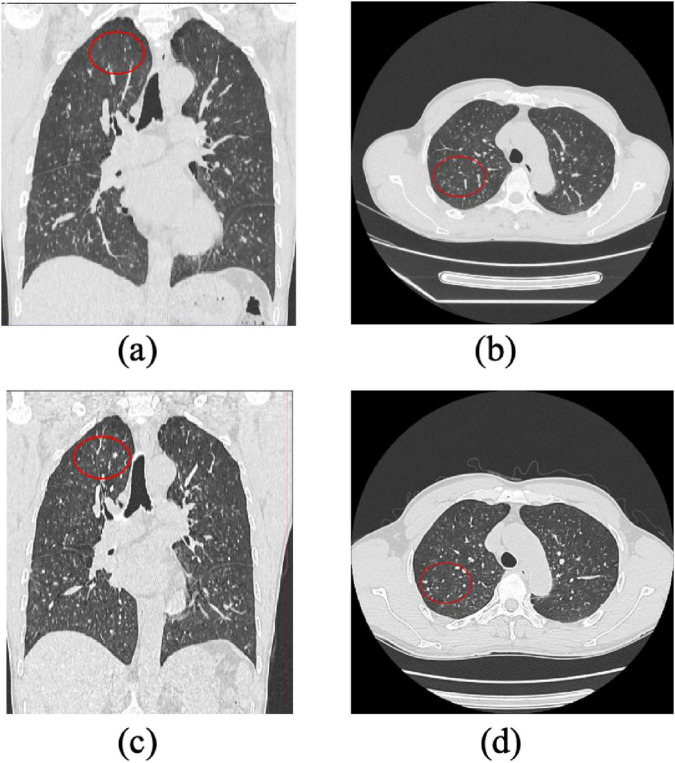
Progression of Pulmonary Lesions in a Stage I Pneumoconiosis Patient: A 1-Year Follow-Up CT Study. **(a)** and **(b)**: Initial CT scans show multiple small high-density nodular opacities and linear fibrotic streaks distributed in the bilateral upper and middle lung fields, primarily affecting the upper lobe segments and lower lobe dorsal segment. **(c)** and **(d)**: Follow-up CT images taken 1 year later reveal an increased number of nodular and fibrotic lesions in the same lung regions, indicating progression of fibrosis. The findings suggest disease advancement over time.

**FIGURE 4:**
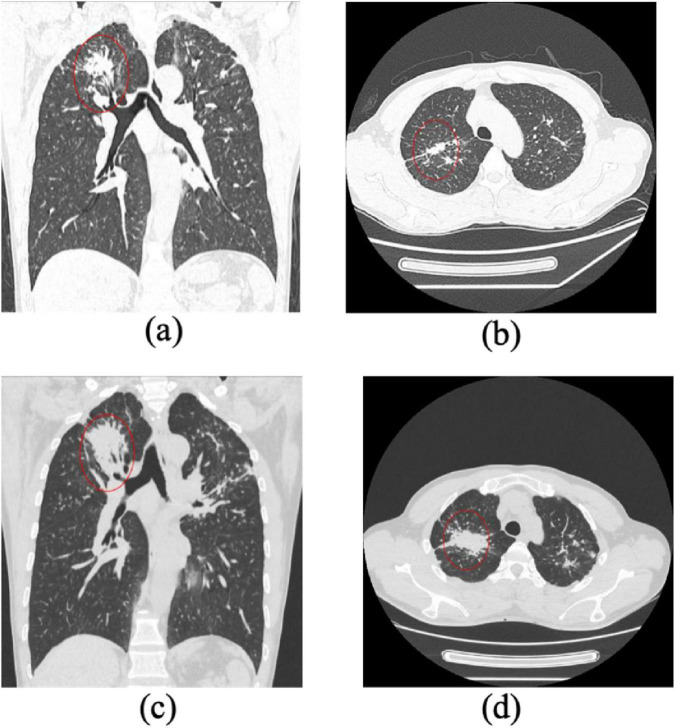
Progression of pulmonary fibrosis in a stage II pneumoconiosis patient: a 1-Year Follow-Up CT Study. **(a,b)** Initial CT scans show large patchy opacities in the apical-posterior segment of the bilateral upper lobes, with a long-axis diameter >2 cm and a short-axis diameter>1 cm. Additionally, multiple scattered high-density nodular opacities are observed in all lung lobes, indicating severe fibrosis. **(c,d)** Follow-up CT images taken 1 year later demonstrate significant progression of fibrotic lesions, with an increase in lesion size and density, suggesting further disease advancement.

Importantly, specific HRCT features such as honeycombing, reticular patterns, and interlobular septal thickening were more prevalent in the progression group, aligning with more advanced fibrotic changes. Although GGO and pleural plaques were present in both groups, their frequency was higher in the progression group, suggesting a potential association with early fibrotic evolution (Figures 3, 4). These observations highlight the complementary diagnostic value of HRCT in conjunction with clinical indicators for the early detection and monitoring of pulmonary fibrosis progression in CWP.

### 3.2 Feature selection and machine learning model development

A total of 851 quantitative radiomic features were extracted from the manually segmented HRCT images of the 297 enrolled CWP patients. These features encompassed first-order statistics, three-dimensional shape descriptors, and multiple texture matrices (GLCM, GLRLM, GLSZM, and NGTDM), enabling a comprehensive representation of parenchymal tissue heterogeneity and fibrotic alterations.

**FIGURE 5:**
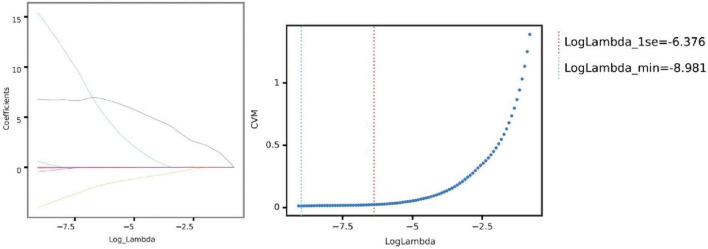
Selection of optimal lambda parameter for LASSO regression in predicting pulmonary fibrosis progression. **(Left)** Coefficient profile plot for the LASSO regression model, showing the trajectory of feature coefficients as the regularization parameter (log Lambda) changes. As the penalty increases, more coefficients shrink toward zero, emphasizing feature selection for model sparsity. **(Right)** Cross-validation curve for the LASSO model, with the mean squared error (CVM) plotted against log Lambda. The vertical dashed lines indicate the optimal Lambda values: the minimum error (left line) and the largest Lambda within one standard error of the minimum (right line). These Lambda values guide feature selection, balancing model complexity and predictive performance.

To address the high dimensionality and potential multicollinearity of the dataset, feature selection was performed using the LASSO regression, a robust method for identifying informative predictors while preventing overfitting. Through LASSO penalization, 19 radiomic features with the highest predictive value for fibrosis progression were retained (Figure 5).

**FIGURE 6:**
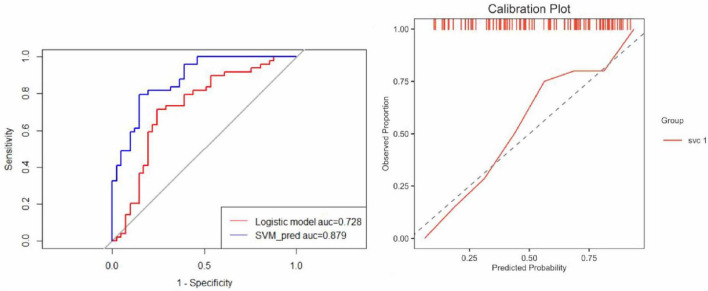
Comparative performance of logistic regression and SVM models. **(Left)** ROC curves illustrate the discrimination performance of the Logistic Regression and SVM models. The AUC for the Logistic Regression model was 0.728, indicating moderate predictive capability, whereas the SVM model achieved a significantly higher AUC of 0.879, demonstrating superior predictive accuracy and robustness. **(Right)** The calibration plot evaluates the agreement between predicted probabilities and observed outcomes. The SVM model calibration curve aligns closely with the ideal diagonal line, reflecting excellent calibration with no evidence of overfitting and reliable predictive performance.

These selected features were subsequently used to construct predictive models using two ML algorithms: LR and SVM. LR was chosen for its interpretability and probabilistic output, while SVM was employed for its robustness in handling non-linear decision boundaries. Model performance was evaluated based on the area under the receiver operating characteristic curve (AUC), complemented by calibration curves to assess the agreement between predicted and observed outcomes (Figure 6). Both LR and SVM models demonstrated satisfactory predictive performance, with minimal evidence of overfitting.

These findings affirm the utility of radiomics as a non-invasive biomarker strategy for tracking fibrotic progression in CWP. The ability to extract and apply high-dimensional image-derived information-coupled with ML -based classification-provides a promising approach for risk stratification and early intervention planning.

### 3.3 Model construction and performance evaluation

**FIGURE 7:**
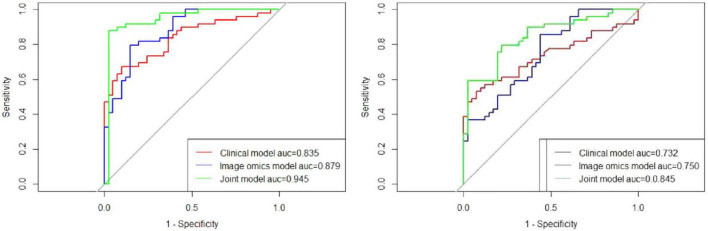
Comparative predictive performance of clinical, radiomic, and joint models. **(Left)** ROC curves demonstrate the predictive performance of the clinical, radiomic, and joint models in the training cohort. The AUC values were 0.835, 0.879, and 0.945 for the clinical, radiomic, and joint models, respectively, highlighting the superior performance of the joint model. **(Right)** In the test cohort, the ROC curves show AUC values of 0.732 for the clinical model, 0.750 for the radiomic model, and 0.845 for the joint model, further validating the enhanced predictive capability of the joint model compared to the individual models.

Following the feature selection and radiomic biomarker construction, three different models were developed for predicting pulmonary fibrosis progression in CWP patients: the clinical model, the radiomic model, and the joint model. The clinical model relied solely on clinical features such as DCH, lung function measures (e.g., FVC, FEV1, FEV1/FVC ratio), and other clinical biomarkers. The radiomic model, in contrast, used only the selected radiomic features derived from the CT images. Finally, the joint model integrated both clinical and radiomic features in an attempt to combine the strengths of both data types for more accurate predictions. The performance of these models was assessed using ROC curves for both the training cohort and test cohort. As shown in Figure 7, the joint model consistently outperformed both the clinical and radiomic models in terms of predictive accuracy.

In the training cohort, the AUC for the ROC curve was 0.835 for the clinical model, 0.879 for the radiomic model, and 0.945 for the joint model. These results indicate that while both clinical and radiomic models demonstrated satisfactory performance, the joint model achieved the highest predictive accuracy, suggesting that the integration of clinical and radiomic data provides superior results. Similarly, in the test cohort, the clinical model had an AUC of 0.732, the radiomic model had an AUC of 0.750, and the joint model reached an AUC of 0.845. These findings further validate the robustness of the joint model across different cohorts, demonstrating its potential for reliable prediction of pulmonary fibrosis progression in CWP patients. The AUC values for all three models are detailed in Table 3, providing a comprehensive overview of their performance.

**TABLE 3:** Comparative predictive performance of clinical, radiomic, and joint models in training and test cohorts.

	Training cohort			Test cohort		
Metric	Clinical model	Radiomic model	Joint model	Clinical model	Radiomic model	Joint model
AUC (95% CI)	0.835 (0.753∼0.918)	0.879 (0.809∼0.948)	0.945 (0.889∼1.000)	0.732 (0.627∼0.838)	0.750 (0.650∼0.850)	0.845 (0.765∼0.925)
Accuracy	76.3%	70.8%	92.1%	88.3%	68.4%	80.9%
Sensitivity	67.3%	79.6%	87.8%	53.1%	85.7%	79.6%
Specificity	90.2%	85.4%	97.6%	92.6%	56.1%	78.0%

**FIGURE 8:**
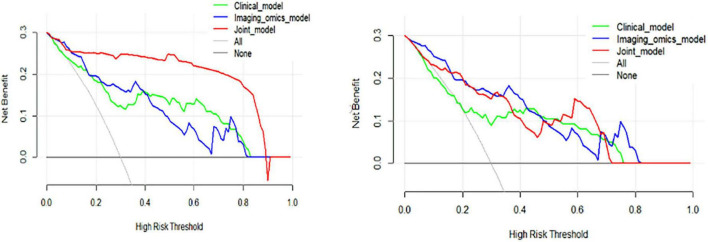
Decision curve analysis (DCA) curves of the training and test cohorts. **(Left)** DCA curves for the training cohort illustrate that the clinical model consistently outperformed the radiomic model, while the joint model provided the highest net benefit across a range of threshold probabilities, demonstrating its superior predictive utility. **(Right)** Similarly, in the test cohort, the clinical model surpassed the radiomic model, with the joint model achieving the highest net benefit, further reinforcing its robust performance and practical value in decision-making scenarios.

In addition to ROC curve analysis, DCA was performed to assess the clinical utility of the models by evaluating the net benefits of using each model at different threshold probabilities. In the training cohort, DCA showed that the clinical model performed better than the radiomic model, but the joint model outperformed both, demonstrating the highest net benefit. This trend was consistent in the test cohort, where the joint model continued to show superior performance over the clinical and radiomic models. The results of the DCA are illustrated in Figure 8, reinforcing the clinical relevance of the joint model for guiding treatment decisions in CWP patients.

Overall, these results demonstrate that combining radiomic signatures with clinical indicators yields more accurate and clinically meaningful predictions of pulmonary fibrosis progression than either approach alone. The joint model offers a promising tool for early risk assessment, potentially enabling timely therapeutic intervention in high-risk CWP patients.

## 4 Discussion

### 4.1 Integration of radiomics and clinical features enhances prediction

High-resolution computed tomography remains central to the diagnosis and monitoring of ILDs, including pneumoconiosis-related pulmonary fibrosis. Owing to its excellent spatial resolution and sensitivity to fibrotic changes, HRCT allows accurate assessment of disease extent and progression (23, 24). Quantitative image analysis, enabled by radiomics, further augments this process by extracting high-dimensional features that capture tissue heterogeneity, structural alterations, and fibrotic remodeling not readily discernible by human readers (25, 26).

In our study, we extracted 851 radiomic features from baseline HRCT images of coalworkers, and employed a LASSO-based feature selection strategy to reduce redundancy and identify 19 predictive features. These were combined with pulmonary function indicators (FVC, FEV1, FEV1/FVC) to build a multi-modal ML model, which demonstrated strong predictive performance for fibrosis progression. The joint model achieved an AUC of 0.945 in the training cohort and 0.845 in the test cohort, outperforming radiomic-only and clinical-only models. DCA further confirmed its clinical net benefit across a wide range of threshold probabilities. These results suggest that integrating radiomics with clinical features can enhance early risk assessment in pneumoconiosis, providing a non-invasive and scalable tool to support individualized monitoring and early intervention strategies.

### 4.2 Clinical and biological interpretability of selected features

To enhance the transparency and clinical relevance of our predictive model, we further explored the interpretability of the radiomic features selected during model construction. Among the 19 features retained after LASSO selection, many were texture-related metrics derived from GLCM, GLSZM, and first-order statistics. These features quantify intrapulmonary heterogeneity, density distribution, and spatial arrangement-attributes known to correlate with pathological alterations in fibrotic lung diseases (27, 28). For instance, features such as GLCM entropy, cluster shade, and GLSZM small area emphasis reflect increased structural complexity and textural irregularity, which are characteristic of fibrotic remodeling in pneumoconiosis (9). First-order features like skewness and kurtosis may reflect asymmetric density distribution, potentially related to the uneven deposition of fibrotic tissue and alveolar collapse (29).

Although these quantitative features do not directly correspond to conventional radiological signs, their patterns are consistent with the fibrotic processes observed in histopathology and high-resolution imaging studies (30). Their selection suggests that the model captures biologically meaningful signals beyond what can be perceived visually, offering potential non-invasive biomarkers for disease progression. Moreover, the combination of radiomics with clinical indicators such as FVC, FEV1, and FEV1/FVC enhances the robustness and interpretability of the model (31). Pulmonary function tests reflect physiological impairment due to restrictive ventilation, while radiomic features reflect structural deterioration. Their joint use aligns with the multidimensional nature of fibrosis progression (32).

This interpretability not only reinforces confidence in the model’s predictions but also promotes clinical acceptance by providing insight into the underlying biological rationale (33). In a broader context, our study demonstrates the feasibility of using explainable radiomic signatures to supplement functional metrics, potentially improving early detection, treatment planning, and monitoring in occupational lung diseases (34).

### 4.3 Calibration and threshold optimization for clinical use

To ensure the clinical applicability of our ML models, we conducted a detailed evaluation of calibration performance and optimized the decision threshold for risk stratification (35). Calibration analysis, which assesses the agreement between predicted probabilities and observed outcomes, revealed that the SVM-based radiomic model demonstrated excellent alignment with the ideal calibration line in the test cohort, indicating reliable probability estimates and minimal overfitting (36). This is visually supported by the calibration curve (Figure 6, right), which confirms the model’s strong probabilistic performance and its potential to inform clinical decision-making. Furthermore, threshold optimization was performed to determine a clinically relevant decision cutoff for distinguishing high-risk patients with likely fibrosis progression from those with stable disease (37, 38). Using Youden’s Index derived from ROC analysis, the optimal threshold was identified for the joint model, which achieved the highest predictive accuracy among all models (39). At this threshold, the model balanced sensitivity and specificity, providing a practical decision point for early intervention planning (40).

Although Brier scores were not explicitly reported in the results, the close calibration of the SVM and joint models suggests a low average prediction error, further supporting their utility in real-world settings (41). These findings underscore the model’s readiness for integration into routine risk assessment workflows, offering clinicians a non-invasive, data-driven tool to support personalized surveillance strategies in patients with CWP (42).

### 4.4 Addressing potential confounders and sampling bias

Despite the promising performance of our integrated radiomics-clinical model in predicting radiological progression in patients with CWP, several potential confounders and biases must be acknowledged (43). Firstly, this study was conducted retrospectively at a single institution with a limited sample size, which may have introduced selection bias. Patients included had relatively complete follow-up data and high-quality HRCT imaging, potentially excluding more severe or comorbid cases and thus limiting the generalizability of the model to the broader CWP population (44).

Secondly, although our model incorporated key clinical predictors-such as age, LDH, and pulmonary function indices (FEV1/FVC and FVC% pred)-there may be unmeasured confounders that also influence disease progression. For example, occupational exposure intensity, smoking status, and comorbid pulmonary conditions (e.g., COPD, silicosis) were not fully accounted for due to data constraints (45). Additionally, manual segmentation of the lungs and lesions, while performed by experienced radiologists with good interobserver agreement, introduces some subjectivity that could affect the consistency of radiomic feature extraction, especially in borderline cases or early-stage disease.

Lastly, the class imbalance between progression and stable groups, though addressed by down-sampling and cross-validation, could still bias the model’s learning process (46). Furthermore, the lack of external validation in independent cohorts remains a key limitation (47, 48). Nevertheless, this study represents an important step toward integrating quantitative imaging biomarkers and clinical parameters to non-invasively assess early progression in CWP. Future multicenter, prospective studies with standardized imaging and a broader spectrum of clinical variables are needed to validate and refine this predictive approach (49, 50).

### 4.5 Conclusion and future directions

The present study constructed a combined predictive model based on HRCT radiomic features and pulmonary function indicators, which showed high accuracy, good calibration, and favorable clinical utility in identifying patients at risk of pulmonary fibrosis progression. These findings provide a novel and non-invasive approach for risk stratification in occupational pulmonary disease. However, several limitations should be acknowledged. Firstly, the study was conducted at a single center, which may restrict generalizability due to variations in CT acquisition protocols and population characteristics (51). Secondly, the model has not yet undergone external validation on an independent dataset. While internal validation yielded consistent results across training and test sets, future work should involve multi-center, prospective validation to confirm robustness (52, 53). Thirdly, the retrospective nature of the study may introduce bias in data collection and outcome classification. Although image acquisition was standardized, longitudinal follow-up data were limited (54). Future studies should adopt a prospective design with repeated imaging and clinical evaluation to enable dynamic prediction modeling (55). Lastly, while radiomic features provide valuable information, their direct biological correlates remain partially understood. Ongoing efforts in imaging-pathology correlation and multi-omics integration are essential to further refine feature selection and enhance clinical adoption (56, 57).

In conclusion, our study presents a predictive framework combining HRCT-derived radiomic features and pulmonary function data to identify patients at risk of pulmonary fibrosis progression among coalworkers. The integrated model demonstrated high predictive accuracy, reliable calibration, and favorable clinical utility (58). With further external validation and real-world testing, such a tool may support early intervention and individualized disease monitoring in occupational respiratory health (59).

## Data Availability

The original contributions presented in this study are included in this article, further inquiries can be directed to the corresponding authors.

## Footnotes

1
https://www.slicer.org/

2
https://www.statsape.com/
